# Host-mycobiome metabolic interactions in health and disease

**DOI:** 10.1080/19490976.2022.2121576

**Published:** 2022-09-24

**Authors:** Neelu Begum, Azadeh Harzandi, Sunjae Lee, Mathias Uhlen, David L. Moyes, Saeed Shoaie

**Affiliations:** a Centre for Host-Microbiome Interactions, Faculty of Dentistry, Oral & Craniofacial Sciences, King’s College London, London, UK; b Science for Life Laboratory, KTH–Royal Institute of Technology, Stockholm, Sweden

**Keywords:** Mycobiome, microbiome, metabolism, host-mycobiome interaction, systems biology, secondary metabolism

## Abstract

Fungal communities (mycobiome) have an important role in sustaining the resilience of complex microbial communities and maintenance of homeostasis. The mycobiome remains relatively unexplored compared to the bacteriome despite increasing evidence highlighting their contribution to host-microbiome interactions in health and disease. Despite being a small proportion of the total species, fungi constitute a large proportion of the biomass within the human microbiome and thus serve as a potential target for metabolic reprogramming in pathogenesis and disease mechanism. Metabolites produced by fungi shape host niches, induce immune tolerance and changes in their levels prelude changes associated with metabolic diseases and cancer. Given the complexity of microbial interactions, studying the metabolic interplay of the mycobiome with both host and microbiome is a demanding but crucial task. However, genome-scale modelling and synthetic biology can provide an integrative platform that allows elucidation of the multifaceted interactions between mycobiome, microbiome and host. The inferences gained from understanding mycobiome interplay with other organisms can delineate the key role of the mycobiome in pathophysiology and reveal its role in human disease.

## Introduction

Until recently, fungal infections, unlike bacterial and viral infections, had been largely neglected despite being a significant cause of global morbidity and mortality.^1,2^ The importance of fungi in the host has been largely overlooked as the number of fungal genes in the gut is relatively low compared to bacterial genes, as reported by the Human Microbiome Project (HMP).^3^ With the advent of high throughput sequencing technologies, our knowledge of the bacterial communities of the microbiome has expanded exponentially, along with our understanding of their potential roles in disease pathology. In contrast, the mycobiome (fungal community) study remains in its formative years.^4–6^ To date, there are substantially fewer studies focused on fungi within the microbiome due to difficulties in nucleic acid extraction, rudimentary fungal genome annotations and limited bioinformatic tools to analyse these data or established standards. Early studies using culture-dependent studies suggested that only 70% of the population harboured a fungal population.^7^ A total of 267 fungal taxa have been identified in the human gut mycobiome by several studies. However, the validity of some of these fungal species (e.g. *Penicillium*) is unclear as they cannot be grown under human gut conditions in the laboratory.^8^ The long-term residence of fungi in the gut microbiome is thus still the subject of debate as the longevity of fungal species within a mycobiome is undetermined.^8^ Further, fungal genes within the microbial metagenome have been estimated to only make up 0.1% of the total microbial genes.^9^ However, the importance of fungi to the host cannot be determined by measures of the overall relative abundance of their genetic material within the microbiome. Production of metabolites and small molecules by fungi potentially has a more significant impact in the microbiota and host compared to bacterial production, owing to the greater fungal biomass and associated higher occupation of surface area, however, this remains largely unproven.^9–11^ A summary table of mycobiome studies associated with dysbiosis highlights the increasing relevance of fungi (Table 1).

**Table 1.** The effect of fungal species in human health. The table indicates the fungal species causing diseases with references.

Species	Disease	Reference
*Candida spp.; Cryptococcus spp., S. cerevisiae*	Liver disease	Bajaj et al., 2013; Hwang et al., 2014; Yang et al., 2017; Fernandez et al., 2017
*S. cerevisiae; Candida spp.; Malassezia spp.*	Irritable Bowel Disease	Ott et al., 2008; Sokol et al., 2017; Nishida et al., 2018;
*Candida spp.; Malassezia spp.*	Crohn’s Disease	Barclay et al., 1992; Poulain et al., 2009; Standaert-Vitse et al., 2009; Hoarau et al. 2016; Liguori et al., 2016; Limon et al., 2019
*Aspergillus sp.*	Cystic Fibrosis	Knutsen et al., 2003; Horre et al., 2004; Bakare et al., 2003; Delhaes et al., 2012; Willger et al., 2014;
*Aspergillus spp.; Epicoccum nigrum; Wallemia sebi*	Lung Diseases	Denning et al., 2011; Knutsen et al., 2012; Fairs et al., 2010; Yan et al 2009; McCarthy and Walsh, 2017
*Aspergillus spp.*	Chronic Obstructive Pulmonary Disease (COPD)	Garcia-Vidal et al., 2008; Guinea et al., 2010; Huerta et al., 2014; Molinos-Castro et al., 2020
*Candida spp.; Aspergillus spp.*	Nosocomial Blood Infections	Wisplinghoff et al., 2004; Morgan et al., 2005; Pfaller et al., 2007; Wang et al., 2020; Sfeir et al., 2020
*Candida spp.*	Vaginal Infection	Taylor et al., 2005; Barousse et al., 2007; Sobel, 2007; llkit and Guzel, 2011
*Cryptococcus spp.*	Meningitis	Gottfredsson and Perfect, 2000; Lui e tal., 2012; Blatzer et al., 2020; Spencer et al., 2020
*Candida spp.*	Oral infection	Barclay et al., 1992; Poulain et al., 2009; Standaert-Vitse et al., 2009; Hoarau et al. 2016; Liguori et al., 2016; Limon et al., 2019
*Candida spp.; Aspergillus spp.; Coccidioides posadasii;*	Neurological Infection	Sharma et al., 1997; Chopra et al., 2006; Thurtell et al., 2013; Suresh, 2015; Pisa et a;., 2015; Benito-Leon and Laurence, 2017; Forbes et al., 2019
*Candida spp.*	Autism spectrum	Strati et al., 2017; Forbes et al., 2019
*S.cerevisiae; Candida spp.*	Schizoprenia	Severance et al., 2016; Severance et al., 2017; Cihakova et al., 2019; Zhang et al, 2020

The variation in species seen within the mycobiome is attributable to dietary changes, skin contamination, drug therapy, geographical location and oral hygiene.^8,12,13^ There are, however, some common features to the different mycobiome communities. *Candida* and *Saccharomyces* are the most common fungal genera in the gut, with their presence detected from infancy, indicating they are likely to be permanent colonisers.^3,4,8,12,14–17^ *Aspergillus* and *Penicillium* genera are less common, indicating the transient nature of these genera, whilst reports of *Mucor, Cladosporium* and *Cyberlindera* presence are inconsistent. The disparate nature of fungal community structure is due to culture-dependent and -independent approaches lacking gold standards.^18^ For example, varying extraction methods and primer selection can skew mycobiome analyses. In essence, there is a lack of reference gene catalogues and bioinformatic tools for consistent taxonomic profiling and reconstitute fungal metagenome species.

Although increasingly being seen as important, investigations of the impact of the mycobiota on both host and microbiome are in their infancy. In particular, our understanding of host-fungal metabolic interactions is limited in both health and disease. Although host-microbiome metabolic interactions have been studied, further development is needed in understanding the impact of fungal biochemical pathways and their downstream metabolites in these interactions.^19^ In this review, we explore the role of fungal metabolism in human disease, and this will help us understand the extent of how mycobiome interacts with its environment. This review will go onto explore the impact of secondary metabolites and how we can use systems-level approaches to explore this deeper level of functioning of the mycobiome for therapeutic use.

## Mycobiome and importance in health and disease

In the last decade, fungal infection research has exploded, focussing on the host immune response towards these microbes, including adaptive and innate immunity.^20,21^ Even though fungal disease leads to inflammation, the mechanisms at mucosal surfaces are not well elucidated.^2^ However, the host immune response and subsequent imbalance of homeostatic microbial communities can lead to dysbiosis. Dysbiosis is the unfavourable changes/imbalance in the community of organisms within the microbiota, leading to the progression of the disease. This term is highly used in microbiome research and infers misunderstanding as the biological context is undetermined as healthy microbiota remains unknown and not specific to the presence of a particular genus or community distribution.^22^ Detailed information of data source was tabulated for *in vivo* and *in vitro* mycobiome studies (Table 2).

**Table 2.** Mycobiome tabulated data types. The table indicates references with fungal data types for discerning fungal genera.

Reference	Data type
Han, S. H. et al. Analysis of the skin mycobiome in adult patients with atopic dermatitis. Exp. Dermatol. 27, 366–373 (2018).	Human data
Mukherjee, P. K. et al. Oral Mycobiome Analysis of HIV-Infected Patients: Identification of Pichia as an Antagonist of Opportunistic Fungi. PLoS Pathog. 10, (2014).	Human data (saliva)
Hoffmann, C. et al. Archaea and Fungi of the Human Gut Microbiome: Correlations with Diet and Bacterial Residents. PLoS ONE 8, (2013).	Human data (stool)
Nash, A. K. et al. The gut mycobiome of the Human Microbiome Project healthy cohort. Microbiome 5, 153–153 (2017).	Human data (HMPS stool)
Ghannoum, M. A. et al. Characterization of the oral fungal microbiome (mycobiome) in healthy individuals. PLoS Pathog. 6, (2010).	Human data (oral rinse)
Drell, T. et al. Characterization of the Vaginal Micro- and Mycobiome in Asymptomatic Reproductive-Age Estonian Women. PLoS ONE 8, (2013).	Human data
David, L. A. et al. Diet rapidly and reproducibly alters the human gut microbiome. Nature 505, 559–563 (2014).	Human data (stool)
Mar Rodríguez, M. et al. Obesity changes the human gut mycobiome. Sci. Rep. 5, 14,600–14,600 (2015).	Human data (stool)
Sokol, H. et al. Fungal microbiota dysbiosis in IBD. Gut 66, 1039–1048 (2017)	Human data (stool)
Trojanowska, D. et al. The role of Candida in inflammatory bowel disease. Estimation of transmission of C. albicans fungi in gastrointestinal tract based on genetic affinity between strains. Med. Sci. Monit. Int. Med. J. Exp. Clin. Res. 16, CR451-457 (2010).	Human data (biopsy)
Ott, S. J. et al. Fungi and inflammatory bowel diseases: Alterations of composition and diversity. Scand. J. Gastroenterol. 43, 831–841 (2008	Human data (biopsy)
Alonso, R. *et al*. Fungal infection in patients with Alzheimer’s disease. *J. Alzheimers Dis. JAD* **41**, 301–311 (2014)	Human data (biopsy)
Alonso, R., Pisa, D., Aguado, B. & Carrasco, L. Identification of Fungal Species in Brain Tissue from Alzheimer’s Disease by Next-Generation Sequencing. *J. Alzheimers Dis. JAD* **58**, 55–67 (2017)	Human data (biopsy)
Severance, E. G. et al. Gastrointestinal inflammation and associated immune activation in schizophrenia. Schizophr. Res. 138, 48–53 (2012)	Human data (blood)
Severance, E. G. *et al*. Candida albicans exposures, sex specificity and cognitive deficits in schizophrenia and bipolar disorder. *Npj Schizophr*. **2**, 1–7 (2016)	Human data (blood)
Severance, E. G. et al. Probiotic normalization of Candida albicans in schizophrenia: A randomized, placebo-controlled, longitudinal pilot study. Brain. Behav. Immun. 62, 41–45 (2017).	Human data (blood)
Hoarau, G. et al. Bacteriome and mycobiome interactions underscore microbial dysbiosis in familial Crohn’s disease. mBio 7, (2016).	Human data (stool)
Aykut, B. et al. The fungal mycobiome promotes pancreatic oncogenesis via activation of MBL. Nature 1–4 (2019) doi:10.1038/s41586-019-1608-2.	*in vivo (mice)*
Harriott, M. M. & Noverr, M. C. Candida albicans and Staphylococcus aureus Form Polymicrobial Biofilms: Effects on Antimicrobial Resistance. Antimicrob. Agents Chemother. 53, 3914–3922 (2009)	*in vitro*
Guinan, J., Wang, S., Hazbun, T. R., Yadav, H. & Thangamani, S. Antibiotic-induced decreases in the levels of microbial-derived short-chain fatty acids correlate with increased gastrointestinal colonization of Candida albicans. Sci. Rep. 9, 8872–8872 (2019).	*in vivo (mice)*
Nguyen, L. N., Lopes, L. C. L., Cordero, R. J. B. & Nosanchuk, J. D. Sodium butyrate inhibits pathogenic yeast growth and enhances the functions of macrophages. J. Antimicrob. Chemother. 66, 2573–2580 (2011).	*in vitro*
Noverr, M. C. & Huffnagle, G. B. Regulation of Candida albicans Morphogenesis by Fatty Acid Metabolites. Infect. Immun. 72, 6206–6210 (2004).	*in vitro*
García, C. et al. The Human Gut Microbial Metabolome Modulates Fungal Growth via the TOR Signaling Pathway. mSphere 2, e00555-17 (2017)	*in vitro*
Baltierra-Trejo, E., Sánchez-Yáñez, J. M., Buenrostro-Delgado, O. & Márquez-Benavides, L. Production of short-chain fatty acids from the biodegradation of wheat straw lignin by *Aspergillus fumigatus*. Bioresour. Technol. 196, 418–425 (2015)	*in vitro*
Borges, F. M. et al. Fungal Diversity of Human Gut Microbiota Among Eutrophic, Overweight, and Obese Individuals Based on Aerobic Culture-Dependent Approach. Curr. Microbiol. 75, 726–735 (2018).	Human data (stool)
Auchtung, T. A. et al. Investigating Colonization of the Healthy Adult Gastrointestinal Tract by Fungi. mSphere 3, e00092-18 (2018).	Human data (saliva, stool HMP)
Strati, F. et al. Age and Gender Affect the Composition of Fungal Population of the Human Gastrointestinal Tract. Front. Microbiol. 7, (2016)	Human data (stool)
Qin, J. et al. A human gut microbial gene catalogue established by metagenomic sequencing. Nature 464, 59–65 (2010)	Human data (stool)
Raimondi, S. et al. Longitudinal Survey of Fungi in the Human Gut: ITS Profiling, Phenotyping, and Colonization. Front. Microbiol. 10, (2019)	Human data (stool)
Botschuijver, S. et al. Intestinal Fungal Dysbiosis Is Associated With Visceral Hypersensitivity in Patients With Irritable Bowel Syndrome and Rats. Gastroenterology 153, 1026–1039 (2017).	Human data (stool)
Iliev, I. D. et al. Interactions between commensal fungi and the C-type lectin receptor Dectin-1 influence colitis. Science 336, 1314–1317 (2012)	*in vivo (mice)*
Kaur, J. et al. “Pseudomonas aeruginosa inhibits the growth of Scedosporium aurantiacum, an opportunistic fungal pathogen isolated from the lungs of cystic fibrosis patients.” *Frontiers in microbiology*, 866. (2015).	in vitro (human sputum)

*in vitro (lab experiments), in vivo (animal experiments), in silico (computational analysis) and human data (clinical sample collection)

Fungal by-products have diverse uses, including agricultural supplements, food preservation, environmental farming, biotechnology and medicines.^10,23^ Routine consumption of fungal derivatives has inevitably resulted in changes to our ecosystem and adaptation of the human mycobiome. For example, fungal species have been used as probiotics, with *Saccharomyces boulardii* currently being used as a probiotic for the treatment of gastrointestinal disorders.^24^ Meanwhile, *S. cerevisiae* isolated from food sources inhibits the adhesion, filamentation and biofilm formation of multiple *Candida* species.^25^ Additionally, exposure to environmental factors including age, gender, diet, host genetics, therapeutic intervention and host lifestyle affect individual mycobiome composition.^5,8,14,26^ Diets with high carbohydrates can drive the increased gut presence of *Candida* species.^14,27,28^ Likewise, obesity is associated with an increased abundance of Ascomycota and a parallel decrease in gut mycobiome richness.^29^

Fungal dysbiosis potentially affects a significant portion of the population, being implicated in irritable bowel syndrome (IBS) and Crohn’s disease (CD) and a direct contributing factor in IBD^30^ (see Table 1). For example, greater fungal diversity occurs in CD patients compared to healthy controls,^31,32^ whilst *C. albicans* overgrowth in the gastrointestinal (GI) tract is a contributing factor to IBD onset.^31–36^ Further, a shift in *Ascomycota* and *Basidiomycota* proportions potentially drives inflammatory processes in IBD.^31,32,37^ The distal effect of mycobiome dysbiosis is chronic immune-mediated inflammation and appears to be related to central nervous system (CNS) infection associated with neurological diseases.^38–40^ Fungal products have been found in Alzheimer patients,^41^ whilst another study confirmed the presence of 14 fungal species, including *Malassezia, Candida, Alternaria* and *Botrytis*,^42^ in different brain regions of Alzheimer’s patients.^43^ Similarly, fungi have been associated as triggers of autoimmunity and multiple sclerosis,^44^ whilst the presence of *C. albicans* and *S. cerevisiae* antibodies in schizophrenia patients has been linked to the gastrointestinal disorder, supporting the hypothesis of a gut-brain axis.^5,38,39,45^

Mycobiome dysbiosis is a predisposing factor in other diseases as well.^37,40^ For example, the mycobiome is associated with cancer development and progression,^46^ where *Malassezia spp*. are increased in pancreatic ductal adenocarcinoma (PDA) in an *in vivo* (murine) study.^46^ In contrast, *Candida tropicalis*, another commensal fungus, is associated with slower or lack of tumour development.^46^ This study, among others, indicates that mycobiome composition is paramount to host health and disease status. However, specific fungal species do not exclusively drive these interactions. In order to survive, fungi adapt to their local environment; this includes adjusting to acidic environments through morphology switching and metabolic plasticity. Biofilms are a classic example of fungi changing their growth pattern in response to host responses and environmental cues. Fungi-microbe interactions play a key role in adaptation. Fungi cooperate with bacteria to increase fungal biofilm potential, resulting in significant increases in the severity of inflammatory diseases – most notably in IBD.^37^ Alternatively, *Pichia spp*. interaction with *Candida* results in the reduction of biofilm due to competition in the environment.^47^ Thus, mycobiome composition plays a significant role in the maintenance of the homeostatic relationship within the microbiota. Determining whether fungal-bacterial-host relationships drive compositional changes and whether controlling each element in these networks could potentially prevent disease is a fundamental goal for future microbiome research.

### Mycobiome and it’s environment

The interplay between the mycobiome and host immune responses involves physical interactions at the cell surface, metabolic reprogramming, regulatory pathways, and secretion of small molecules. Exposure to host responses and environmental stressors provides a platform for metabolic plasticity to manipulate host interactions and promote invasion^48^ or modulate immunological responses.^49^ These host immune responses to fungal infection have been reviewed extensively elsewhere.^20,50–54^ To creates symbiotic relationship with microbiota for host immune-tolerance, there is a positive exchange of compounds and nutrients. With evidence of fungi producing vitamins and fatty acids,^55–57^ there is an opportunity and potential for these exchanges with the mycobiome that remains to be investigated.

Microbial metabolism, including fungal metabolism, has been associated with driving inflammation, various diseases and even cancer progression.^58–60^ For instance, short-chain fatty acids (SCFA), tryptophan and branched-chain amino acids have all been identified as playing a role in immune and homeostatic responses.^61^ Short-chain fatty acids (SCFAs) available within the host are necessary for regulating some fungal growth as well as modulating immune responses. Fungi are capable of producing vitamins, notably including vitamin D and B6, that can impact the immune system.^62–66^ The different fungal moieties, such as cell wall polysaccharides and melanin,^67^ drive metabolic reprogramming of innate immune cells to confer protective inflammation and activate antimicrobial defences.^68–70^ Equally, contact with host cells drives shifts in fungal metabolism.^52^ For instance, host recognition of ß-glucan from fungal cell walls reprogrammes macrophage glucose metabolism towards aerobic glycolysis, promoting more rapid ATP creation.^67^ Importantly, however, *C. albicans* undergoes a similar metabolic reprogramming, meaning that local glucose is rapidly depleted, leading to the premature death of macrophages.^52^ As *C. albicans* has a significant degree of metabolic plasticity, it can then revert to using alternative carbon sources to survive. Melanin produced by *Aspergillus fumigatus* is recognised by macrophages via MelLec (a C-type melanin-sensing receptor) via calcium sequestering in phagosomes, leading to activation of antifungal defences within the host.^71^ *A. fumigatus* responds further by reducing melanin for fungal persistence, leading to conidia germination and increased glycolysis allows metabolic repurposing of macrophage response for effective fungal defence.^70^ Hosts defensive metabolic responses and the parallel evasive mechanistic pathways in fungi demonstrate a need to understand metabolic networks in host mycobiome in health and disease.^52^

### Bacteriome-mycobiome interaction

In a series of animal model studies, the interaction between host-mycobiome and fungus-bacterium has been linked to the health status of the host (expansion of the *in vivo* data set sources is listed in Table 2).^37,72–74^ The interaction of fungi and bacteria occurs on several different levels. Physical contact can occur between members of microbial communities leading to the exchange of secreted products and communication between the two cell surfaces. Cell signalling between microbes also provides regulation and trade of metabolites and genetic materials through different mechanisms, such as horizontal gene transfer, signal transduction and interaction with the host.^75,76^ This exchange allows for an interplay between microbes that can maintain the ecosystem’s resilience or drive a dysbiosis state. This interplay within the host means there is a knock-on effect for the host, potentially resulting in disease pathology. For example, *C. tropicalis* is significantly more abundant in Crohn’s disease (CD) patients in comparison to non-CD relatives.^37^ This increased abundance correlates with increased abundance of some bacterial species and related improved biofilm formation and disease severity. These interactions can potentially be bi-directional. For example, *Escherichia coli, Serratia marcescens*, and *Candida tropicalis* together exhibit enhanced biofilm formation, giving a thicker, more robust film.^37^ Furthermore, the production of bile acids (e.g. cholic acid) from the microbiota and environment have been shown to promote the growth and morphogenetic plasticity of *C. albicans*,^77^ whilst hexadecanedioic and caproic acids have demonstrated antifungal activity.^29^

Recently, multiple studies have begun to elucidate how our bacteriome and mycobiome interact with each other and the contribution of these interactions to human health and disease. In particular, the bacteriome and mycobiome can alter each other’s composition.^28^ For example, competition between bacteria and fungi leads to stunted *Candida* growth.^78^ The release of various compounds can drive these interactions. For instance, *Pseudomonas aeruginosa* releases bacterial compounds called phenazines, in particular pyocyanin, that suppress *A. fumigatus, C. albicans* and *Cryptococcus neoformans* growth in the lungs of cystic fibrosis patients (*in vitro;* Table 2).^79^ Nor are these interactions purely suppressive. Dimethyl sulphide, a volatile compound released by strains of *P. aeruginosa* derived from human sputum samples, stimulates the growth of *A. fumigatus* within *in vitro* experiments.^80^ These bacterial-produced compounds demonstrate the varied effects of secondary metabolites produced by the bacteriome on the mycobiome. Moreover, *P. aeruginosa* has been shown to attach to *C. albicans* hyphae, killing them via type IV pili.^81^ Similarly, *Staphylococcus aureus* and *C. albicans* are frequently co-isolated in immunocompromised patients.^82,83^ Serum analysis showed *S. aureus* has preferential adherence to *C. albicans* driving hyphae formation. Interestingly, *S. aureus* formed microcolonies with a halo formation on the surface of *C. albicans* biofilm, resulting in an increase in vancomycin resistance.^84^ *C. albicans* hyphae initiates innate responses in attracting phagocyte cells for transporting the fungi into a protected intracellular host environment. *S. aureus is* shown to take advantage of this by escaping intracellular killing and disseminating to cause sepsis.^85^

Alteration of the bacteriome composition drives diverse changes in metabolite levels in the gut and effects on the mycobiome. For example, antibiotic-treated mice have increased levels of *C. albicans* in their gut.^77^ Whilst it could be argued that this is purely due to an increase in the available nutrients, this is unlikely to be the sole reason, given the lack of presence of this species in mice under normal conditions. There is evidence of SCFA release by bacteria incurring modifications in host transcription activation,^86–88^ signalling^89,90^ and chromatin changes^91^ in the host. The increased fungal presence may be due to bacterial species reduction resulting in diminished SFCA production, giving rise to changes in pH. Therefore, the changes in bacterial abundance due to antibiotics drive shifts in gut bacteriome composition and abundance, altering cross-talk with the mycobiome. There is further potential for fungi to interact with SCFAs. *Aspergillus* can produce SCFAs from aromatic compounds biodegradation. *A. fumigatus* degrades these compounds to produce acetic acid, among others.^92^ Investigation of the effects of SCFAs on *C. albicans* in the presence of bacteria found they control yeast growth.^93^ Further investigation revealed that butyrate and lactic acid inhibit hyphal germination^94,95^ whilst sodium butyrate increases phagocytic macrophage rates along with increasing production of nitric acid.^96^ SCFAs also induce biofilm formation in *C. albicans*, decreasing its metabolic activity.^77^ Acetate exhibited the most potent inhibitory effects on *C. albicans*, directly preventing germ tube formation and adherence independent of pH.^77^ These studies indicate that microbially-produced SCFAs (from either bacteriome or mycobiome) affect both confirmation and community structure of the mycobiome, potentially leading to dysbiosis.

### Mycobiome metabolism

Fungi produce an expansive variety of metabolites with varied impacts on their survival, as well as host and microbiota interactions. For example, the fungal metabolite N-acetyl-L-glutamic acid has hypotensive effects.^29^ The natural ability of fungi to produce this cornucopia leads to ripple effects in their environment.^97^ The metabolic activity of fungal communities has a pivotal role in microbiome modulation, contributing to disease pathogenesis and pathology. Fungi can catabolise a panoply of different substrates, including carbohydrates, amino acids, lipids, proteins, and vitamins utilising both primary and secondary metabolism. Primary metabolism is involved in the growth and development of the cell, whilst secondary metabolism increases fungal viability in the environment. Fungi produce a variety of secondary metabolites, including acids, toxins, polyols and sugars that mediate cross-talk and are considered a by-product of other reactions.^98^

Secondary metabolites are key by-products of primary reactions and play a crucial role in shaping the interaction within a niche. In the elucidation of enzymatic processes, fungal secondary metabolites have been classed into several groups depending on the biosynthesis of these products. The most notable involves polyketide synthases (PKS), non-ribosomal peptides synthases (NRPS), fatty acid derivatives, terpenoids, and steroids.^99–102^ Polyketide (PKS) class consists of derivatives of fatty acids and the output of acetyl CoA pathways that allow for various structures and functions.^102,103^ Non-ribosomal peptides (NRPS) are derived from amino acids and cannot perform RNA transcription.^99,101,102,104^ Terpenes class are generated from terpene synthases, a product of the mevalonic acid pathway and produced as steroid compounds.^105^ Terpenoids are a modified class of terpenes. Dimethyallyl tryptophan (DMATs) class is comparable to the ergot alkaloid class and are usually pharmacologically active compounds responsible for producing indoles.^106–108^ As the detection of growing numbers of metabolites progresses, methods in finding genes responsible for secondary metabolites are also being developed. These genes are called biosynthetic gene clusters (BGCs) and are based on the chromosomal arrangement of the synthesis of secondary metabolites. These BGCs ranges from smaller clusters relating to synthases to a larger cluster of genes that include transcription factors, as detailed in Keller et al. (2019). These genes are globally regulated by transcriptional activity, not all necessarily from within the cluster, thus indicating that transcription and epigenetic activation of BGCs is dependent on external factors. These factors include environmental stimuli, metal presence, nutritional availability, temperature, pH and light, triggered by regulatory pathways.^76^

**Figure 1.**
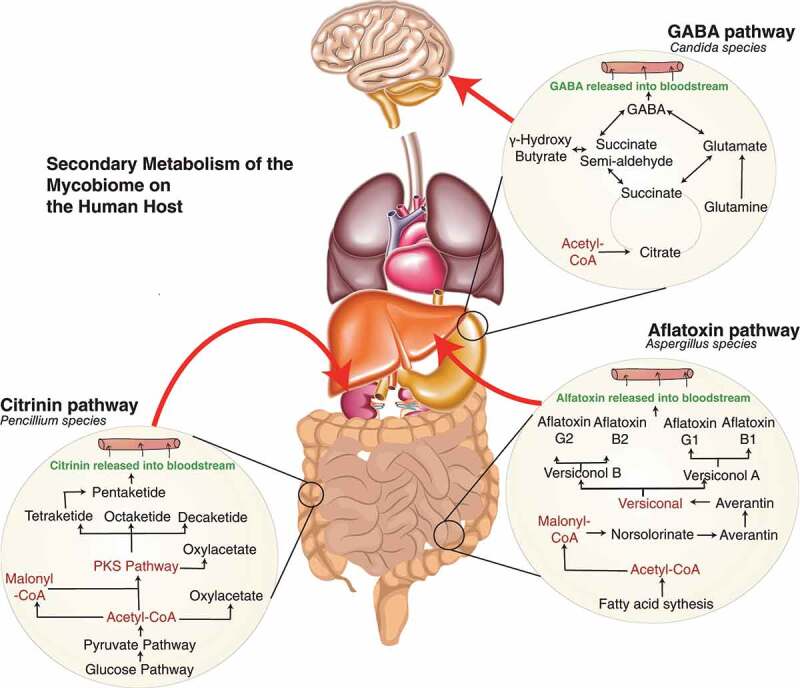
Fundamental metabolic interactions between host-mycobiome in health and disease. A. indicates *Candida* contribution to the production of γ-amino butyric acid (GABA) through the tricarboxylic acid cycle to produce succinate may perceptively occur during systemic infection. GABA has a role in dampening the effect of the nervous system, and generally, deficiency involves epilepsy.^109^ Indirectly targeting mycobiome in specific regions such as *Candida* species can have a better effect than providing analogues of GABA that has been identified to lead to anxiety, stress and seizures. Exploring this metabolic pathway of the mycobiome, especially targeting the succinate pathway, the production of semi-aldehydrase could lead to alteration of disease with better regulation from the microbiota, as alternatively, high levels of GABA have been linked to Alzheimer’s disease.^110^ B. *Penicillium* species’ ability to produce the mycotoxin called citrinin is toxic to cause renal nephrosis.^111–113^ Drugs have been developed to inhibit the effect of citrinin in hepatoma, and several animal studies have shown the benefit.^114^ Ostry et al., 2013 have highlighted the dangerous effect of citrinin in dietary sources.^115^ There has been no established link of mycobiome being the contributor of toxin in humans and whether diverting the polyketide pathway can reduce renal cell toxicity. C Aflatoxin is a common secondary metabolite of the *Aspergillus* family. It has been associated with cancer morbidity and inducing tumour suppressor gene p53.^116–119^ The potential effect of the presence of *Aspergillus* needs to be investigated and whether manipulating the metabolic pathway can give a better indicator of being able to reverse the effects of aflatoxin by reducing the levels of the malonyl-coA pathway. All these potential pathways give us a clear indication of how the study of the metabolism of the mycobiome is essential in understanding pathogenesis across a wide breadth of diseases.

The mycobiome includes metabolic processes with an extensive pool of secondary metabolite production that can be key in driving or preventing a pathogenic switch. For instance, metabolites and small molecules can activate the host immune system via recognition of foreign material or the release of toxins, driving inflammatory responses. Potential effects of known metabolites produced by *Candida, Penicillium* and *Aspergillus* species and their possible metabolic pathways have been illustrated in Figure 1, demonstrating a likely impact on the host during infection.

Secondary metabolites can have a knock-on effect on host metabolism, and this has been used extensively for therapeutic effects (Table 3). One metabolite isolated from *Aspergillus* species is naptho-γ-pyrones. This natural metabolite has various biological effects, including anti-bacterial and anti-oxidant activity.^120–124^ The wide variety of fungal metabolite functions makes them ideal targets for therapeutic use. This can be done by targeting metabolic pathways to derive benefits. For example, by driving immunometabolic shifts that affect glucose competition and redirecting pathways for cancer treatment.^125^ As well as direct impacts on the host whether as colonisers or during infection, these metabolites are ideal potential drugs for treating the microbiome directly. For example *Penicillium* produces a mycotoxin called citrinin that has antibiotic properties against bacteria and can have a toxic effect on kidney and liver.^126–128^ Secondary metabolites can thus conceivably both provide diagnostic markers for the detection of disease and manipulate metabolic pathways, altering host health status. System biology contributes useful tools in investigating metabolic pathways in fungi in the context of the human mycobiome.

**Table 3.** **Highlight the effect of metabolites on the host**. Identification of a wide variety of secondary metabolites from various fungal species, the class grouping attributed to the metabolite, based upon the synthesis, fungal species responsible for chemically engineering the metabolite and the effect on the human host. The properties include anti-bacterial activity to cancerous effect. The extended table includes the effect on the environment, marine biology and produce. *IL = interleukin ** blue highlight indicates human effect.

Metabolite	Class	Fungi	Activity	Reference
Averufin	PKS	*Aspergillus versicolor*	Anti-bacterial activity	Miao et al., 2012; Goyal, 2016; Yu, 2012
Aflatrem	DMATs	*Aspergillus flavus*	Acute neurotoxic effects	Hoffmeister, 2016; Valdes et al., 1985
Agrocalvin-I	Alkaloids	*Pencillium sp.*	Induce changes in GABA providing inhibitory effect of CNS, anti-bacterial and anti-tumor activites	Selala et al., 1989; Griffin et al., 2013; Niehaus etl al., 2016; Kumar et al., 2018
Ascomycone A (6-deoxyfusarubin)	PKS	*Biatriospora sp.*	Cytotoxic	Stodulkova et al., 2015; Goyal, 2016
Asperdiazapinones	Alkaloids	*Aspergillus sp. PSU-RSPG185*	Anti-fungal, cytotoxic and insecticidal activities	Rukachaisirikul et al., 2013; Yin et al, 2009; Siddiquee, 2018
Asperochrins	PKS	*Aspergillus oryzae*	Antibacterial acitivity; inhibitory cidal activity against aquatic species	Liu et al., 2015; Siddiquee, 2018
Chanoclavine	Alkaloids	*Pencillium spp.*	Induce changes in GABA providing inhibitory effect of CNS	Selala et al., 1989; Griffin et al., 2012; Xu et al., 2015; Kumar et al., 2018;
Citrinin	PKS	*Pencillium spp.*	Anti-bacterial activity; Toxins damaging organs (renal disease)	Hetherington and Raistrick, 1931; Subramani et al., 2013; Zain et al., 2011; Samson et al., 2011a; Bouslimi et al., 2008; Goyal, 2016
Cladosin C	PKS	*Cladosporium sphaerospermum*	Anti-viral activity	Wu et al., 2014; Goyal, 2016
Deoxynivalenol (vomitoxin)	NRPS	*Fusarium graminearum; Fusarium culmorum*	Immunosupressive activity; potential suspectible to viral and bacterial infections	Marasas et al, 1984; Bondy and Peska, 2000; Palazzini et al., 2016; Moss, 2011
Elymoclavine	DMATs	*Aspergillus spp.*	Inhibitory effect of CNS	Robbers, 1979; Hoffmeister, 2016
Festuclavines	Alkaloids	*Pencillium spp.*	Induce changes in GABA providing inhibitory effect of CNS, anti-bacterial and anti-tumor activites	Selala et al., 1989; Griffin et al., 2014; Kumar et al., 2018
Flaviphenalenones	PKS	*Aspergillus flavipes*	Anti-malarial cytotoxic and antimicrobial activity	Nazir et al., 2015; Gutierrez et al., 2013; Elsebai et al., 2011; Siddiquee, 2018
Fumagillin	Terpene	*Aspergillus fumigatus*	aNtibiotic treatment for protozoa, analogues treatment for angiogensis and supress tumour growth	Schenk et al, 1953; hanson and Elbe, 1949; Ingber et al., 1990; Molina et al., 2002; Moss, 2011; Siddiquee, 2018
Isochanoclavine	Alkaloids	*Pencillium spp.*	Induce changes in GABA providing inhibitory effect of CNS	Selala et al., 1989; Griffin et al., 2016; Kumar et al., 2018; Rabha and Jha, 2018
Isofellutanine	Alkaloids	*Pencillium spp.*	Anti-bacterial and anti-tumor activites	Jouda et al., 2016; Kumar et al., 2018
Naptho-γ-pyrones	PKS	*Aspergillus brasiliensis*	Anti-tumoral, anti-bacterial, anti-fungal	Song et al., 2004; Koyama et al., 1988; Samson et al., 2007; Siddiquee, 2018; Se-Kwon Kim, 2013
Neoechinulin A	Alkaloids	*Eurotium spp.*	Anti-inflammatory effect	Kim et al., 2013; Goyal, 2016; Chen et al., 2015
Pestalotiopsone A	PKS-NRPS	*Pestalotiopsis spp.*	Anti-bacterial activity	Hemberger et al., 2013; Goyal, 2016; Kumar et al., 2018

### Systems and synthetic biology approach to elucidate interactions

**Figure 2.**
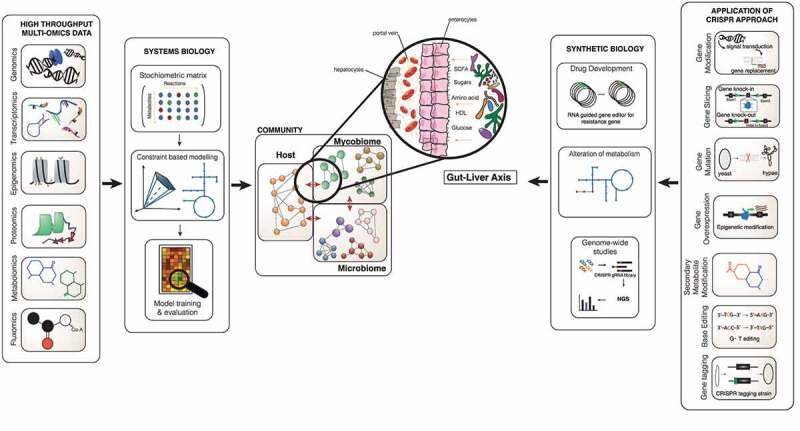
System and synthetic biology approach in investigating mycobiome’s important role in the gut-liver axis. This image shows the interaction of the microbiome-mycobiome with the host cells in the gut, translocation of intermediaries through the blood, and prospectively leading to the composition of the liver environment to change. The biological knowledge from the collection of omics data such as proteome, metabolome and transcriptome generates information that can be integrated into functional mathematical models. The structural data is placed into the stoichiometric matrix and after several steps of adding biochemical information the draft of the GSMM can be reconstructed and further validated for new biological interpretation and predictions. Different constraint can be applied on the GSMMs to investigate the models for novel discovery. CRISPR as one of the gene editing approaches in validating predictions based on GSMM; these can be done using the CRISPR-cas9 system to perform genome modification such as gene slicing, gene mutation, secondary metabolite modification, base editing, and gene editing tagging and gene overexpression, down-regulation methods. The application can be used in tackling drug resistance, alteration of the metabolic pathway with the possibility of amending toxic pathways in pathogenesis, rationalising gene essentiality and genome-wide screening. The application of these potential methods in assessing the mycobiome community and particularly within the gut-liver axis.

To date, mycobiome studies have primarily been grounded in culture-based techniques that are hostage to the diverse requirements of each community member, meaning many fungi remain undetected and undiscovered.^4^ The development of molecular techniques such as 18S ribosomal RNA and inter transcribed spacer (ITS) sequencing has profoundly impacted the detection of fungi and the characterisation of the mycobiome. However, these techniques are limited in understanding the mycobiome community’s overall structure, function, and dynamic existence on a large-scale platform.^129^ Many mycologists face the obstacle of limited bioinformatic tools and databases available compared to bacteriologists. With improving next generation sequencing (NGS) technologies and lower costs, multi-omics data generation is increasing rapidly. Multi-omics data includes metagenomics, transcriptomics, proteomics, metabolomics and fluxomics that impart in-depth information that has enabled us to learn about microbiota interactions (Figure 2).^130–132^ Each multi-omics dataset provides a different view on either single species or communities of varied microbes.^133,134^ Experimental studies and multi-omics data generate abundant information; however, we currently lack the ability to fully integrate this information to give us a systematic overview. Individual data have so far failed to elucidate the interactions involved in fungi-fungi, fungi-bacteria and fungi-host relationships. Systems biology, by its holistic approach and mathematical models, provides a computational platform to integrate the different multi-omics datasets, presenting a better structure to understand the relationship between gene, protein and metabolites, and additionally to explore the interactions between organisms in a complex community.^33,135–137^ Among the mathematical models as the core of the systems biology approach, genome-scale metabolic models (GSMM) has proven to enable the genotype-phenotype relationships through a series of connected gene-protein-reaction links, providing a powerful predictive toolkit.^135^ GSMM have been applied in different microbial and human studies as a discovery platform to propose novel markers, treatments and bioproduction enhancement.^138–141^ GSMM predictions and discovery can be further evaluated and validated using synthetic biology, such as CRISPR-cas systems (Clustered Regularly Interspace Short Palindromic Repeat) for genome editing to test and manipulate molecular and metabolic pathways.^142^

The *S. cerevisiae* model is the benchmarking fungal GSMM with a comprehensive network structure including the 3D structure and protein kinetics.^143^ This GSMM is a reference model used to generate the scaffold of other fungal species models.^30^ Other well-constructed fungal reference models that have been generated include *Scheffersomyces stipitis*,^144^ *C. glabrata*^145,146^ and *Pichia pastoris*.^147^ Fungal GSMM are mainly used to inform the metabolic engineering experiments, increase the efficiency of the process and markers, such as modelling yeast suggested methods for increasing vanillin production by 5-fold.^148^ Similarly, the model’s biological interpretation of *Yarrowia lipolytica* metabolic pathways identified that this fungal species is an ideal producer of di-carboxylic acid using a lipid pathway.^149^

The application of GSMM technologies to the human microbiome has identified cross-feeding of metabolites between host and microbiota in health and disease.^139^ This has led to a recent increase in studies predicting biological behaviour, the use of biotics for metabolic diseases, personalised medicine, and drug targets for pathogens.^149–154^ Modelling has implicated the production of SCFAs from microbial fermentation in the host in CD aetiology; thus, adjusting diet can control the presence of SCFAs in patients and modulate disease.^155^ Exploring IBD compared to healthy cohorts showed an increase in vitamin metabolism in IBD patients, whilst a closer inspection of species in the microbiome using GSMMs unveiled vitamin and biotin producing gut organisms in those suffering from IBD,^156^ whilst using modelling of the microbiome in neurological diseases including Parkinson’s Disease^157–160^ and Alzheimer’s disease^161,162^ has identified microbiome perturbations, causing flux changes in pathways and production of SCFAs that ultimately potentially cause disease. Studies on community modelling of the microbiome predict metabolic interactions between species with the overall metabolic contribution of each species determined.^163–165^ Research in this area remains in its infancy as the development of algorithms and tools used to explore community modelling continues, but the potential is beyond doubt.

As there are several available fungal GSMM, they could be implemented in studying the mycobiome to elucidate the complex interactions between mycobiome, bacteriome and host, thereby explaining the metabolic role of fungi in disease pathophysiology. Exploring the mycobiome with GSMM will significantly improve our understanding of fungal interactions with the host in health and disease. Synthetic biology can then be used to validate and assess genetic circuits and metabolic frameworks for complex interactions. Recently, CRISPR-based systems for repression and activation in *C. albicans* have been used to investigate its regulatory system in the clinical context.^166^

The difficulties lie in exploiting fungal models and synthetic biology due to annotation accuracy with high plasticity of metabolic pathways, diverse morphology, and evolutionary adaptive traits. There is a lagging development of fungal GSMM and applying CRISPR system in clinical and healthcare settings due to a lack of quantitative tools to study fungal genomes a lack of annotation and validation in the model. The global overview provided by this approach offers the potential to drive strategies to manipulate the mycobiome to make predictions, test hypotheses, propose clinical areas for manipulation, identify new diagnostics target areas with better understanding, and discover novel therapeutic targets.^135,136,167^

## Conclusion

Understanding the behaviour of fungi, both individually and as a community, is essential in elucidating the role of the mycobiome in health and disease. Untethering the distinct metabolic interactions and effects of secondary metabolism provide an opportunity for disease intervention, determining the point at which dysbiosis arises, identifying biomarkers for better diagnostics and novel therapeutic targets. Metabolism is a holistic and integrative subject that combines genetics, molecular pathways, signalling and environmental factors. Multi-disciplinary tools and methods such as system biology are needed to develop *in silico* models as a predictive tool to validate current datasets and knowledge. In doing so, we will generate a platform to answer how mycobiome metabolism affects the host in health and disease.

## Background information – what is a genome-scale metabolic model (GSMM) and CRISPR

Genome-scale metabolic models (GSMM) are a systematic and curated method to establish genotype-phenotype relationships. GSMM aim to bridge together the complex network of genes, reactions and thousands of metabolites *in silico* while sustaining full metabolic flux functionality of the system. The functionality of a model refers to the natural ability of the model to undertake reactions, a realistic rate of energy consumption, rate of energy production and apply physio-chemical laws and environmental input to create a system that is true-to-life.^168^ The conversion of the reactions into a stoichiometric matrix allows mathematical inferences to integrate data into a predictive biological framework called constraint-based modelling.^169^ This developmental process of integration requires automated and manual curation for efficient quality standards. The GSMM community has developed a MEMOTE suite package to ensure the models’ standardisation and functional feasibility.^170^ With synthetic biology approach of genome engineering techniques pave the way for validating fungal species’ biological mechanisms underlying these systematic alterations in response to different environmental changes such as response to anti-fungal drug and the host organism’s reaction during fungal infection (Martins-Santana et al., 2018).^142^ Figure 2 demonstrates the step-by-step inferences for creating a GSMM and synthetic biology to determine the interaction of mycobiome within the community.

The creation of a GSMM includes two methods: firstly, a top-down approach, which integrates experimental data into a mathematical model. This method makes it possible to create a network of reactions and metabolic outputs through pathways. The study of metabolism can provide more accuracy and specificity with further predictive analysis and assessment. Secondly, the bottom-up approach includes defined knowledge and information sourced from readily available studies and the input of current literature (Machado et al., 2018).^171^ The current approach to reconstructing GSMM combines top-down and bottom-up approaches using automated tools such as the RAVEN toolbox (Agren et al., 2013).^172^ GSMM can be used in constraint-based modelling to predict the flux distribution and secretion of metabolites within the organism under certain constraints such as the intake of substrates and governing specific objective functions such as growth, thereby providing information that would be difficult to generate in *vitro* experiments (Bordbar et al., 2014).^169^ As of 2019, there are reconstructed GSMM for 434 bacteria, 40 archaea and 117 eukaryotes (Gu et al., 2019).^30^ The majority of these GSMM are used in the simulation of a single organism. However, their use can go further to study the metabolic cross-talk of microbial communities.

CRISPR (Clustered Regularly Interspace Short Palindromic Repeat-associated (Cas) is a robust and customised method of gene editing which is adapted from the immune mechanism in bacteria and archaea against viral (bacteriophage) invasion (Mojica et al. 2005).^173^ What makes Type-II CRISPR-Cas9 a well-established CRISPR approach in the genome-editing field is the ability of manipulation at genome level using just chimeric single guide RNA, making double-strand breaks to insert-deletion gene (indel) using cas9 nuclease, followed by homologous/non-homologues recombination and multiple genes indel (Pourcel et al. 2005; Brouns et al. 2008).^109–119,174,175^

## Supplementary Material

Supplemental Material
