# Supplementary material for: Host-mycobiome metabolic interactions in health and disease
Source: Gut Microbes. 2022 Sep 24;14(1):2121576. doi: 10.1080/19490976.2022.2121576 (PMC9519009; doi:10.1080/19490976.2022.2121576)
Supplement: Supplemental Material [file KGMI_A_2121576_SM6482.zip › Begum_et_al_supplementary_information1.docx]

**Supplementary information1 – What is a genome-scale metabolic model (GEMs) and CRISPR**

Genome-scale metabolic models (GEMs) are a systematic and curated method to establish genotype-phenotype relationships. GEMs aim to bridge together the complex network of genes, reactions and thousands of metabolites *in silico* while sustaining full metabolic flux functionality of the system. The functionality of a model refers to the natural ability of the model to undertake reactions, realistic rate of energy consumption, rate of energy production and apply physio-chemical laws and environmental input to create a system that is true-to-life ^1^. The conversion of the reactions into a stoichiometric matrix allows mathematical inferences to integrate data into a predictive biological framework called constraint-based modelling ^2^. This developmental process of integration requires automated and manual curation for efficient quality standards. The GEM community has developed a MEMOTE suite package to ensure the models' standardisation and functional feasibility ^3^. With synthetic biology approach of genome engineering techniques pave the way for validating fungal species’ biological mechanisms underlying these systematic alterations in response to different environmental changes such as response to anti-fungal drug and the host organism’s reaction during fungal colonisation ^4^. Figure 2 demonstrates the step-by-step inferences for creating a GEM and synthetic biology to determine the interaction of mycobiome within the community.

The creation of a GEM includes two methods: firstly, a top-down approach, which integrates experimental data into a mathematical model. This method makes it possible to create a network of reactions and metabolic outputs through pathways. The study of metabolism can provide more accuracy and specificity with further predictive analysis and assessment. Secondly, the bottom-up approach includes defined knowledge and information sourced from readily available studies and the input of current literature ^5^. The current approach to reconstructing GEMs combines top-down and bottom-up approaches using automated tools such as the RAVEN toolbox ^6^. GEMs can be used in constraint-based modelling to predict the flux distribution and secretion of metabolites within the organism under certain constraints such as the intake of substrates and governing specific objective functions such as growth, thereby providing information that would be difficult to generate in *vitro* experiments ^2^. As of 2019, there are reconstructed GEMs for 434 bacteria, 40 archaea and 117 eukaryotes ^7^. Many of these GEMs are used in the simulation of a single organism. However, their use can go further to study the metabolic crosstalk of microbial communities.

CRISPR (Clustered Regularly Interspace Short Palindromic Repeat-associated (Cas) is a robust and customise method of gene editing which is adapted from immune mechanism in bacteria and archaea against viral (bacteriophage) invasion ^8^. What makes Type-II CRISPR-Cas9 as well-established CRISPR approach in genome editing field is the ability of manipulation at genome level using just chimeric single guide RNA, making double strand breaks to insert-deletion gene (indel) using cas9 nuclease, followed by homologous/non-homologues recombination and multiple genes indel ^9,10^.

The *S. cerevisiae* model is the benchmarking fungal GEM with a comprehensive network structure including 3D structure and protein kinetics ^11^. This GEM is a reference model used to generate the scaffold of other fungal species^7^. Different well-constructed fungal reference model includes *Scheffersomyces stipites* ^12^, *C. glabrata*^13,14^ and *Pichia pastoris* ^15^. Fungal GEMs are mainly used in metabolic engineering, such as yeast modelling investigation suggested method for increasing vanillin production by 5-fold ^16^, biological interpretation of *Yarrowia lipolytica* metabolic pathway from the model identified that this fungi is an ideal producer for di-carboxylic acid using lipid pathway ^17^ and pan-genome analysis of *Malassezia* species to highlight metabolic differences from evolutionary investigations ^18^.

References

1. Norsigian, C. J., Fang, X., Seif, Y., Monk, J. M. & Palsson, B. O. A workflow for generating multi-strain genome-scale metabolic models of prokaryotes. *Nat. Protoc.* **15**, 1–14 (2020).

2. Bordbar, A., Monk, J. M., King, Z. A. & Palsson, B. O. Constraint-based models predict metabolic and associated cellular functions. *Nat. Rev. Genet.* **15**, 107–120 (2014).

3. Lieven, C. *et al.* MEMOTE for standardized genome-scale metabolic model testing. *Nat. Biotechnol.* **38**, 272–276 (2020).

4. Martins-Santana, L. *et al.* Systems and Synthetic Biology Approaches to Engineer Fungi for Fine Chemical Production. *Front. Bioeng. Biotechnol.* **6**, 117 (2018).

5. Machado, D., Andrejev, S., Tramontano, M. & Patil, K. R. Fast automated reconstruction of genome-scale metabolic models for microbial species and communities. *Nucleic Acids Res.* **46**, 7542–7553 (2018).

6. Agren, R. *et al.* The RAVEN Toolbox and Its Use for Generating a Genome-scale Metabolic Model for Penicillium chrysogenum. *PLoS Comput. Biol.* **9**, (2013).

7. Gu, C., Kim, G. B., Kim, W. J., Kim, H. U. & Lee, S. Y. Current status and applications of genome-scale metabolic models. *Genome Biol.* **20**, 121 (2019).

8. Mojica, F. J. M., Díez-Villaseñor, C., García-Martínez, J. & Soria, E. Intervening sequences of regularly spaced prokaryotic repeats derive from foreign genetic elements. *J. Mol. Evol.* **60**, 174–182 (2005).

9. Brouns, S. J. J. *et al.* Small CRISPR RNAs guide antiviral defense in prokaryotes. *Science* **321**, 960–964 (2008).

10. Pourcel, C., Salvignol, G. & Vergnaud, G. CRISPR elements in Yersinia pestis acquire new repeats by preferential uptake of bacteriophage DNA, and provide additional tools for evolutionary studies. *Microbiol. Read. Engl.* **151**, 653–663 (2005).

11. Lu, H. *et al.* A consensus S. cerevisiae metabolic model Yeast8 and its ecosystem for comprehensively probing cellular metabolism. *Nat. Commun.* **10**, 3586 (2019).

12. Caspeta, L., Shoaie, S., Agren, R., Nookaew, I. & Nielsen, J. Genome-scale metabolic reconstructions of Pichia stipitis and Pichia pastoris and in silico evaluation of their potentials. *BMC Syst. Biol.* **6**, 24 (2012).

13. Sohn, S. B., Kim, T. Y., Lee, J. H. & Lee, S. Y. Genome-scale metabolic model of the fission yeast Schizosaccharomyces pombe and the reconciliation of in silico/in vivo mutant growth. *BMC Syst. Biol.* **6**, 49 (2012).

14. Xu, N. *et al.* Reconstruction and analysis of the genome-scale metabolic network of Candida glabrata. *Mol. Biosyst.* **9**, 205–216 (2013).

15. Chung, B. K. *et al.* Genome-scale metabolic reconstruction and in silico analysis of methylotrophic yeast Pichia pastoris for strain improvement. *Microb. Cell Factories* **9**, 50 (2010).

16. Brochado, A. R. *et al.* Improved vanillin production in baker’s yeast through in silico design. *Microb. Cell Factories* **9**, 84 (2010).

17. Mishra, P. *et al.* Genome-scale model-driven strain design for dicarboxylic acid production in Yarrowia lipolytica. *BMC Syst. Biol.* **12**, 12 (2018).

18. Triana, S. *et al.* Lipid Metabolic Versatility in Malassezia spp. Yeasts Studied through Metabolic Modeling. *Front. Microbiol.* **8**, 1772 (2017).
